# Catheter-related bloodstream infection: factors affecting incidence

**DOI:** 10.1186/cc10657

**Published:** 2012-03-20

**Authors:** K Boner, M McGovern, J Bourke, C Walshe, D Phelan

**Affiliations:** 1 Mater Misericordiae University Hospital, Dublin, Ireland; 2 Harvard University, Cambridge, MA, USA; 3 Beaumont Hospital, Dublin, Ireland

## Introduction

Catheter-related bloodstream infection (CRBSI), its associated morbidity, mortality and expense are the most important adverse effect of central venous catheters (CVCs) [1]. The objective of this study of a population in whom the rate of CRBSI fell significantly over 12 years [2] was to evaluate the influence of both patient and CVC factors on CRBSI rates in patients receiving total parenteral nutrition (TPN) in this time.

## Methods

Set in a 525-bed university hospital providing acute and tertiary services. A prospective database was established in 1997, recording data on all patients with CVCs inserted for TPN administration. This database was examined up to 2009 to ascertain the effects of patient and CVC factors on CRBSI.

## Results

During the 12-year study period, 2,573 CVCs were inserted into 1,343 patients and 15,385 CVC days were accumulated. Overall, 13.8% of patients developed CRBSI throughout the study. In terms of patient factors affecting CRBSI rates, CRBSI was increased in patients with longer duration of TPN administration (where each additional day was associated with a relative risk ratio of 1.02, *P *< 0.01), increased numbers of CVCs inserted (where each additional line was associated with a relative risk ratio of 1.21, *P *< 0.01), and use of lipid formulation of TPN (58.9 vs. 49% use was associated with a relative risk ratio of 1.56, *P *< 0.01). Overall 8.6% of CVCs inserted became infected. Hospital location of CVC insertion was an important risk factor for CRBSI. The most common site for insertion was the ICU (almost 40% of CVCs); however, compared to ICU insertion, insertion in the HDU was associated with an increased risk of CRBSI (a relative risk ratio of 1.75, *P *< 0.01), as was insertion in the operating theatre for ward patients (a relative risk ratio of 2.08, *P *< 0.01). CVC maintenance at ward level was associated with increased CRBSI rates, with a relative risk ratio of 2.06 (*P *< 0.01).

## Conclusion

CRBSI occurs commonly in TPN populations, but there are very limited published data as regards incidence or factors affecting incidence in this population. This large study of TPN patients provides prospective analysis of both patient and CVC factors influencing the development of CRBSI for the first time.
